# Reviewer acknowledgement 2014

**DOI:** 10.1186/s12936-015-0656-9

**Published:** 2015-04-11

**Authors:** Marcel Hommel

**Affiliations:** Editor-in-Chief, Malaria Journal, 236 Grays Inn Road, London, WC1X 8HB UK

## Abstract

*Malaria Journal* would like to thank all reviewers who have contributed to the journal in 2014.

**Rosa Abellana**

Spain

**Ishag Adam**

Sudan

**Emily Adams**

United Kingdom

**Yaw Afrane**

Kenya

**Teamur Aghamolaei**

Iran

**Moses Aikins**

Ghana

**Pietro Alano**

Italy

**Sandra Alba**

Netherlands

**Victor Alegana**

Kenya

**Michael Alifrangis**

Denmark

**Stephen Allen**

United Kingdom

**Lionel Almeras**

France

**Haoues Alout**

United States

**Luke Alphey**

United Kingdom

**Sherif Amer**

Netherlands

**Priyanie Amerasinghe**

Sri Lanka

**Judith Anchang-Kimbi**

Cameroon

**Björn Anderstam**

Sweden

**Frank Andrade**

Brazil

**Heitor Andrade**

Brazil

**Katherine T Andrews**

Australia

**Nicholas Anstey**

Australia

**Spinello Antinori**

Italy

**Gifty Dufie Antwi**

Ghana

**Ana Paula Arez**

Portugal

**Emmanuel Arinaitwe**

Uganda

**Alicia Arnott**

Australia

**Elizabeth Ashley**

Thailand

**Alex Asidi**

United Kingdom

**Sarah Auburn**

Australia

**Robert Aunger**

United Kingdom

**Gordon Awandare**

Ghana

**Diego Ayala**

France

**Hamza Babiker**

Oman

**David Bacon**

United States

**Frank Baiden**

Ghana

**Ripley Ballou**

Belgium

**Kristin Banek**

United Kingdom

**Geetha Bansal**

United States

**Lawrence Barat**

United States

**Barbara Barbé**

Belgium

**Karen Barnes**

South Africa

**John Barnwell**

United States

**Manoel Barral-Netto**

Brazil

**Alyssa Barry**

Australia

**Leonardo Basco**

France

**Quique Bassat**

Mozambique

**Ronan Batista**

Brazil

**Kevin Batty**

Australia

**Jake Baum**

United Kingdom

**Jose Manuel Bautista**

Spain

**Raymond (Ray) Beach**

United States

**Lynette Beattie**

Australia

**Hans-Peter Beck**

Switzerland

**Nigel Beebe**

Australia

**Ron Behrens**

United Kingdom

**John Beier**

United States

**Luis Benavente**

United States

**Mark Benedict**

United States

**Adam Bennett**

United States

**Nicole Isabelle Melanie Berens-Riha**

Germany

**Aase Berg**

Norway

**Yngve Bergqvist**

Sweden

**Pedro Berzosa**

Spain

**Nora Besansky**

United States

**Naomi Beyeler**

United States

**Achuyt Bhattarai**

United States

**Peter Billingsley**

United States

**Christophe Biot**

France

**Gretchen Birbeck**

Zambia

**Zeno Bisoffi**

Italy

**Anders Bjorkman**

Sweden

**Justine Blanford**

United States

**Marlene Blöchliger**

Switzerland

**Karin Blomqvist**

United States

**Aneliya Bobeva**

Bulgaria

**Hervé Bogreau**

France

**Scott Bohle**

Canada

**Kalifa A Bojang**

Gambia

**Angela Boland**

United Kingdom

**Alois Bonifacio**

Italy

**Selina Elisabeth Ruth Bopp**

United States

**Steffen Borrmann**

Germany

**Andrea Bosman**

Switzerland

**Ian Boulton**

United Kingdom

**Catherine Bourgouin**

France

**Teun Bousema**

United Kingdom

**Marielle Karine Bouyou-Akotet**

Gabon

**Hannah Bowen**

United States

**Michelle Boyle**

United States

**Zbynek Bozdech**

Singapore

**Bernard Brabin**

United Kingdom

**Patricia Brasil**

Brazil

**Philippe Brasseur**

Senegal

**Paula Brentlinger**

United States

**Valérie Briand**

France

**William Brieger**

United States

**Sébastien Briolant**

France

**Miguel Brito**

Portugal

**William Brogdon**

United States

**Kathrin Buchholz**

Germany

**Pierre Buffet**

France

**Tullu Bukhari**

Netherlands

**Samuel Bumuh**

French Guiana

**Howard Burkom**

United States

**Maria Dorina Bustos**

Philippines

**Jessica Butts**

United States

**Ib Christian Bygbjerg**

Denmark

**Matthew Cairns**

United Kingdom

**Adriana Calderaro**

Italy

**Félix Calderón**

Spain

**Guido Calleri**

Italy

**Ewan Cameron**

United Kingdom

**Ermanno Candolfi**

France

**Jun Cao**

China

**Margareth Capurro**

Brazil

**Jaime Carmona-Fonseca**

Colombia

**Vern Carruthers**

United States

**Keith Carter**

United States

**Leonardo Carvalho**

United States

**Climent Casaals-Pascual**

United Kingdom

**Francesco Castelli**

Italy

**Marcia Castro**

United States

**Carla Cerami**

United States

**Sandra Chang**

United States

**Tracey Chantler**

United Kingdom

**Pablo Enrique Chaparro-Narváez**

Colombia

**Jacques Derek Charlwood**

United Kingdom

**Himanshu Chaturvedi**

India

**Virander Chauhan**

India

**Luis Fernando Chaves**

Japan

**Lin Chen**

United States

**Stella Chenet**

United States

**Qin Cheng**

Australia

**Vanessa Chen-Hussey**

United Kingdom

**Sheleme Chibsa**

Ethiopia

**Moses Chimbari**

South Africa

**Watcharee Chokejindachai**

Thailand

**Virasakdi Chongsuvivatwong**

Thailand

**Gerardo Chowell**

United States

**Riann Christian**

South Africa

**Mary Christman**

United States

**Cindy Chu**

Thailand

**Thomas Churcher**

United Kingdom

**Jérôme Clain**

France

**Vivien Cleary**

United Kingdom

**Melanie Cocco**

United States

**Claudia Codeco**

Brazil

**Maureen Coetzee**

South Africa

**Lauren Cohee**

United States

**Jessica Cohen**

United States

**Justin Cohen**

United States

**Frank Collins**

United States

**Jan Conn**

United States

**Melissa Conrad**

United States

**Lesong Conteh**

United Kingdom

**Jackie Cook**

Tanzania

**Marc Henri Carl Coosemans**

Belgium

**Fabio Costa**

Brazil

**Michel Cot**

France

**Gilles Cottrell**

Benin

**Kevin Couper**

United Kingdom

**Evans Coutinho**

India

**Jonathan Cox**

United Kingdom

**Janet Cox-Singh**

United Kingdom

**Alister Craig**

United Kingdom

**Jakob Peter Cramer**

Germany

**Pedro Cravo**

Brazil

**Andrea Crisanti**

United Kingdom

**Liwang Cui**

United States

**Richard Culleton**

Japan

**Jane Cunningham**

Switzerland

**Prabin Dahal**

United Kingdom

**Johanna Daily**

United States

**John Dame**

United States

**Cyrus Daneshvar**

United Kingdom

**Claudio Tadeu Daniel-Ribeiro**

Brazil

**Charles Dann**

United States

**Ricardo Luiz Dantas Machado**

Brazil

**Bhabani Sankar Das**

India

**Martin De Smet**

Belgium

**J. Brian de Souza**

United Kingdom

**Bart De Spiegeleer**

Belgium

**P.J. de Vries**

Netherlands

**Eric Deharo**

Peru

**Kirk Deitsch**

United States

**Alessandra della Torre**

Italy

**Philippe Deloron**

France

**Michael Delves**

United Kingdom

**Arlene Dent**

United States

**Marcel Deponte**

Germany

**Dominic Dery**

Ghana

**Johannes Dessens**

United Kingdom

**Silvia Di Santi**

Brazil

**Abdoulaye Diabate**

Burkina Faso

**Anelia Dietmann**

Austria

**Angel Dillip**

Tanzania

**Xavier Ding**

Switzerland

**Navid Dinparast Djadid**

Iran

**Abdoulaye Diop**

Senegal

**Oscar Divala**

Malawi

**Armel Djenontin**

Benin

**Abdoulaye Djimdé**

Mali

**Carlota Dobano**

Spain

**Alexander Nii Oto Dodoo**

Ghana

**Gonzalo J Domingo**

United States

**Arjen M Dondorp**

Thailand

**Tom Drake**

United Kingdom

**Chris Drakeley**

United Kingdom

**Michael Duffy**

Australia

**Scott Duke-Sylvester**

United States

**J. Stephen Dumler**

United States

**Linda Duval**

France

**Philip Eckhoff**

United States

**Michael Edstein**

Australia

**Geoffrey Edwards**

United Kingdom

**Timothy John Egan**

South Africa

**Thomas Eisele**

United States

**Iqbal Elyazar**

Indonesia

**Christian Engwerda**

Australia

**Jaran Eriksen**

Sweden

**Marcy Erskine**

Switzerland

**Ananias Escalante**

United States

**Jayashree Ethiraj**

Italy

**Rick Fairhurst**

United States

**Marit Farenhorst**

Netherlands

**Anna Färnert**

Sweden

**Paddy Farrington**

United Kingdom

**Jean-François Faucher**

France

**Nick Feasey**

United Kingdom

**Heather Ferguson**

United Kingdom

**Carmen Fernandez-Becerra**

Spain

**Delmiro Fernandez-Reyes**

United Kingdom

**Sumadhya Fernando**

Sri Lanka

**Marcelo Ferreira**

Brazil

**Pedro Ferreira**

Japan

**David Fidock**

United States

**Ulrike Fillinger**

United Kingdom

**Phil Fischer**

United States

**Lawrence Fleckenstein**

United States

**Isabelle Florent**

France

**Walter Focke**

South Africa

**Cor Fontes**

Brazil

**Filomeno Fortes**

Angola

**Deshka Foster**

United States

**Woodbridge Foster**

United States

**Freya Fowkes**

Australia

**Matthias Frank**

Germany

**John Frean**

South Africa

**Fréderic Ariey**

France

**David Freedman**

United States

**Friedrich Frischknecht**

Germany

**Gabrielle Froberg**

Sweden

**Anne Frosch**

United States

**Torsten Frosch**

Germany

**Hans-Peter Fuehrer**

Austria

**Nancy Fullman**

United States

**Gawrie Nirdoshi Loku Galappaththy**

Sri Lanka

**Clicia Galardo**

Brazil

**Dionicia Gamboa**

Peru

**David Garboczi**

United States

**Mark Gardner**

United Kingdom

**Michelle Gatton**

Australia

**Oumar Gaye**

Senegal

**Timothy Geary**

Canada

**Peter Gething**

United Kingdom

**Najia Karim Ghanchi**

Pakistan

**Gabriella Gibson**

United Kingdom

**Sabine Gies**

Burkina Faso

**Jose Pedro Gil**

Sweden

**Tim Gilberger**

Germany

**Benjamin Gilbert**

Brazil

**Christopher Gill**

United States

**John Gimnig**

United States

**Sophie Githinji**

Kenya

**Sophia Githinji**

Kenya

**Federico Gobbi**

Italy

**Dean Goldring**

South Africa

**Jacob Golenser**

Israel

**Melba Gomes**

Switzerland

**Luciano Gomes**

Brazil

**Paula Gomes**

Portugal

**Raquel Gonzalez**

Spain

**Iveth Jimena González Jiménez**

Switzerland

**Lilia Gonzalez-Ceron**

Mexico

**Catherine Goodman**

Kenya

**Christopher Dean Goodman**

Australia

**Roly Gosling**

United States

**Louis Clement Gouagna**

Reunion

**Fred Gould**

United States

**Ivan Gout**

United Kingdom

**D. Channe Gowda**

United States

**Mark Grabowsky**

United States

**Justin Green**

United Kingdom

**Michael Green**

United States

**Bryan Greenhouse**

United States

**George Greer**

United States

**Philippe Grellier**

France

**Lynn Grignard**

United Kingdom

**Martin Peter Grobusch**

Netherlands

**Andreas H. Groll**

Germany

**Charlotte Gryseels**

Belgium

**Philippe Guérin**

United Kingdom

**Pierre Guillet**

United States

**Sharmini Gunawardena**

Sri Lanka

**David Gurarie**

United States

**Julie Gutman**

United States

**Davidson R Gwatkin**

United States

**Asrat Hailu**

Ethiopia

**Busiku Hamainza**

Zambia

**Davidson Hamer**

United States

**Ubydul Haque**

Bangladesh

**Laura Harrington**

United States

**Caroline Harris**

United Kingdom

**Steven A. Harvey**

United States

**Ian Hastings**

United Kingdom

**Michael Hawkes**

Canada

**Simon Hay**

United Kingdom

**Olle Heby**

Sweden

**Raimund Helbok**

Austria

**Michelle Helinski**

Uganda

**Christoph Josef Hemmer**

Germany

**Ilse Hendriksen**

Netherlands

**Niel Hens**

Belgium

**John Henshall**

Australia

**Manuel W Hetzel**

Switzerland

**Jorg Heukelbach**

Brazil

**Catherine Hill**

United States

**Jenny Hill**

United Kingdom

**Kenji Hirayama**

Japan

**Alexandra Hiscox**

Netherlands

**Eva Maria Hodel**

United Kingdom

**Johan Hoebeke**

Belgium

**Francis W Hombhanje**

Papua New Guinea

**Sandrine Houze**

France

**Rosalind Howes**

United Kingdom

**George Hui**

United States

**Hilary Hurd**

United Kingdom

**Eleanor Hutchinson**

United Kingdom

**Jimee Hwang**

United States

**Mallika Imwong**

Thailand

**Seth Irish**

United Kingdom

**Deus Ishengoma**

Tanzania

**Saadou Issifou**

Gabon

**Thomas** Jaenisch

Germany

**Zairi Jaal**

Malaysia

**Jan Jacobs**

Belgium

**Prasanna Jagannathan**

United States

**Mark James**

United States

**Chris J. Janse**

Netherlands

**Olivia Jansen**

Belgium

**Susan Jarvi**

United States

**Isabelle Jeanne**

Australia

**Lubin Jiang**

China

**Chandy John**

United States

**Caroline Jones**

United Kingdom

**Somchai Jongwutiwes**

Thailand

**Jonathan Juliano**

United States

**Annette Kaiser**

Germany

**Linda Kalilani-Phiri**

Malawi

**Edwin Kamau**

Kenya

**Akira Kaneko**

Sweden

**Osamu Kaneko**

Japan

**Stefan Kappe**

United States

**Marc Karam**

Switzerland

**Simon Kariuki**

Kenya

**Stephan Karl**

Australia

**Gunasekaran Kasinathan**

India

**Harparkash Kaur**

United Kingdom

**Reginald Kavishe**

Tanzania

**James Kazura**

United States

**Joseph Keating**

United States

**Esther Kellenberger**

France

**Gerard Kelly**

Australia

**Kent Kester**

United States

**Jay Keystone**

Canada

**Insaf Khalil**

Denmark

**Wansika Kiatpathomchai**

Thailand

**Solomon Kibret**

Ethiopia

**Albert Kilian**

Spain

**Gerry Killeen**

Tanzania

**Christopher King**

United States

**Anthony Kiszewski**

United States

**Uriel Kitron**

United States

**Immo Kleinschmidt**

United Kingdom

**Daniel Kline**

United States

**Frank Kloprogge**

Thailand

**Tessa Knox**

Switzerland

**Ellen Knuepfer**

United Kingdom

**Fumie Kobayashi**

Japan

**Tamaki Kobayashi**

United States

**Clemens Kocken**

Netherlands

**Lizette Koekemoer**

South Africa

**Hannah Koenker**

United States

**Constantianus J.M. Koenraadt**

Netherlands

**Kanako Komaki-Yasuda**

Japan

**Sashidhara Koneni**

India

**Kwadwo Koram**

Ghana

**Katrin Krõlov**

Estonia

**Andreas Kudom**

Ghana

**Nirbhay Kumar**

United States

**Florian Michael Kurth**

Germany

**Dennis Kyle**

United States

**Payal Laad**

India

**Paul LaBarre**

United States

**Marcus Lacerda**

Brazil

**David Lalloo**

United Kingdom

**Tracey Lamb**

United States

**Gordon Langsley**

France

**David Larsen**

United States

**Miriam Laufer**

United States

**Saranath Lawpoolsri**

Thailand

**Jean-Yves Le Hesran**

United Kingdom

**Arnaud Le Menach**

United States

**Karine Gaelle Le Roch**

United States

**Rosemary Lees**

Italy

**Gilbert Lefevre**

Switzerland

**Tovi Lehmann**

United States

**Audrey Lenhart**

United States

**Didier Leroy**

Switzerland

**Andres G. Lescano**

Peru

**W. Conrad Liles**

United States

**Felix Limbani**

Malawi

**Jessica Lin**

United States

**Steve Lindsay**

United Kingdom

**Megan Littrell**

United States

**Manuel Llinas**

United States

**Neil Lobo**

United States

**Berlin Londono**

United States

**Dinora Lopes**

Portugal

**Stefanie Lopes**

Brazil

**Lena Lorenz**

United Kingdom

**Reik Löser**

Germany

**Valerie R. Louis**

Germany

**Sonia Lourembam**

India

**Andrew Lover**

United States

**Rachel Lowe**

Spain

**Yoel Lubell**

Thailand

**Adrian Luty**

France

**Lucio Luzzatto**

Italy

**Michael Lynch**

Switzerland

**Matthew Lynch**

United States

**Penelope Lynch**

United Kingdom

**Gloria Macassa**

Sweden

**Michael Macdonald**

United States

**Vanessa Machault**

France

**Margaret Mackinnon**

Kenya

**Amanda Maestre**

Colombia

**Alan Magill**

United States

**Pascal Magnussen**

Denmark

**Jagadish Mahanta**

India

**Alex Maier**

Australia

**Mark Maire**

Zambia

**Kathryn Maitland**

United Kingdom

**Silas Majambere**

Tanzania

**Robert Malima**

Tanzania

**Kathleen Maloney**

United States

**Jessica Maltha**

Belgium

**Sylvie Manguin**

France

**Laurens Manning**

Australia

**Jutta Marfurt**

Australia

**Patricia Marin**

Spain

**Michael Marks**

United Kingdom

**Christopher Marquis**

Australia

**Andreas Mårtensson**

Sweden

**Josué Martínez-de la Puente**

Spain

**Pierre Marty**

France

**Irene Misuka Masanja**

Tanzania

**Eduardo Massad**

Brazil

**Don Mathanga**

Malawi

**Graham Matthews**

United Kingdom

**Kai Matuschewski**

Germany

**Richard Maude**

Thailand

**Juergen May**

Germany

**Alfredo Mayor**

Spain

**Wilfred Mbacham**

Cameroon

**Leonard Mboera**

Tanzania

**Charles Mbogo**

Kenya

**Anthony Mbonye**

Uganda

**Andrea McCollum**

United States

**Malcolm McConville**

Australia

**Rose McGready**

Thailand

**George McKerr**

United Kingdom

**Kate McLean**

United States

**Salah Mecheri**

France

**Rajeev Mehlotra**

United States

**Peter Meissner**

Germany

**Paola Mejia**

United States

**Didier Menard**

Cambodia

**Michela Menegon**

Italy

**Alexander Mentzer**

United Kingdom

**Aboma Merdasa**

Sweden

**Martin Meremikwu**

Nigeria

**Steve Meshnick**

United States

**Christian Meyer**

Germany

**Sungano Mharakurwa**

Zambia

**John Miller**

Zambia

**Juan Pablo Millet**

Spain

**Rakesh Mishra**

India

**Saroj Mishra**

India

**Christian Mitri**

France

**Abraham Mnzava**

Switzerland

**Victor Mobegi**

Kenya

**Frank Mockenhaupt**

Germany

**Fabrizio Molteni**

Tanzania

**Malcolm Edward Molyneux**

Malawi

**Devanand Moonasar**

South Africa

**Bruno Moonen**

Kenya

**Brioni Moore**

Australia

**Julie Moore**

United States

**Sarah Moore**

Tanzania

**Cinta Moraleda**

Spain

**Erin Mordecai**

United States

**Benjamin Mordmueller**

Germany

**Marta Moreno**

United States

**Beatriz Mosqueira**

Spain

**William Moss**

United States

**Atis Muehlenbachs**

United States

**Olaf Mueller**

Germany

**Scott Muerhoff**

United States

**Gunter Muller**

Israel

**Sylke Muller**

United Kingdom

**Pie Müller**

Switzerland

**Maria Anice Mureb Sallum**

Brazil

**Sean Murphy**

United States

**Lise Musset**

French Guiana

**Clifford Mutero**

South Africa

**Kesara Na-Bangchang**

Thailand

**M Nacher**

French Guiana

**Elizabeth Nardin**

United States

**Mohandas Narla**

United States

**Daouda Ndiaye**

Senegal

**Penny Neave**

United Kingdom

**Pramod Nehete**

United States

**Paul Newton**

Laos

**Marie Ng**

United States

**Billy Ngasala**

Tanzania

**Corine Awonja Ngufor**

United Kingdom

**Fatima Nogueira**

Portugal

**Gregory Noland**

United States

**Ace North**

United Kingdom

**Francois Nosten**

Thailand

**Rintis Noviyanti**

Indonesia

**Francine Ntoumi**

Congo

**Stewart Nuttall**

Australia

**Myat Htut Nyunt**

Myanmar

**Ulla Obermayr**

Germany

**Christian Ockenhouse**

United States

**Kathryn O'Connell**

Kenya

**Johanna Ohm**

United States

**Emelda Okiro**

Kenya

**Alaide Oliveira**

Brazil

**Rodrigo Oliveira**

Brazil

**Joseli Oliveira-Ferreira**

Brazil

**Shune Oliver**

South Africa

**Piero Olliaro**

Switzerland

**Peter Olumese**

Switzerland

**Judy Omumbo**

Kenya

**Laura O'Reilly**

Switzerland

**Pamela Orjuela-Sanchez**

United States

**Faith Hope Among'in Osier**

Kenya

**Omran Osman**

Sudan

**Mac Otten**

United States

**Richard Martin Oxborough**

United Kingdom

**Luiz Shozo Ozaki**

United States

**Yoshihisa Ozoe**

Japan

**Krijn Paaijmans**

United States

**M. Andreina Pacheco**

United States

**Giacomo Maria Paganotti**

Italy

**Franco Pagnoni**

Switzerland

**Jennifer Palmer**

United Kingdom

**Gregory Park**

United States

**Christian Parobek**

United States

**Pascal Ringwald**

Switzerland

**Mercedes Pascual**

United States

**Manuel Alfonso Patarroyo**

Colombia

**Jaymin Patel**

United States

**Edith Patouillard**

United Kingdom

**Amy Patterson**

United States

**Richard Paul**

France

**Roger Pearson**

Ethiopia

**Koen Peeters Grietens**

Belgium

**Christopher Pell**

Netherlands

**Stephen Pelly**

South Africa

**Melissa Penny**

Switzerland

**Javier Perez-Tris**

Spain

**Kristina Persson**

Sweden

**Eskild Petersen**

Denmark

**Stefan Peterson**

Sweden

**Johannes Pfeil**

Germany

**Souly Phanouvong**

United States

**Stephen Phillips**

United Kingdom

**Stephane Picot**

France

**Susan Pierce**

United States

**Patricia Pietrantonio**

United States

**David Pigott**

United Kingdom

**Dylan Pillai**

Canada

**Patrice Piola**

Madagascar

**Catherine Pitt**

United Kingdom

**Katherine Plewes**

Thailand

**Nicolas Pocquet**

France

**Adrian Martin Pohlit**

Brazil

**Spencer Polley**

United Kingdom

**Marco Pombi**

Italy

**Marinete Povoa**

Brazil

**Stephen Poyer**

Kenya

**Jetsumon Prachumsri**

Thailand

**Gabriele Pradel**

Germany

**Bruno Pradines**

France

**Surendra Kumar Prajapati**

India

**Lilian Pratt-Riccio**

Brazil

**Ric Price**

Australia

**Bobbi Pritt**

United States

**Miguel Prudencio**

Portugal

**Wendy Prudhomme O'Meara**

Kenya

**Justin Pulford**

Papua New Guinea

**Rachel Pullan**

United Kingdom

**Chaturong Putaporntip**

Thailand

**Brandon Pybus**

United States

**Neils Quashie**

Ghana

**Martha Quinones**

Colombia

**G-Halli Rajasekariah**

Australia

**Rajendranath Ramasawmy**

Brazil

**Michael Ramharter**

Gabon

**Manoranjan Ranjit**

India

**Hilary Ranson**

United Kingdom

**Srikanta Rath**

India

**Julian C Rayner**

United Kingdom

**Linda Reiling**

Australia

**Richard Reithinger**

United States

**Edmond Remarque**

Netherlands

**Franck Remoue**

France

**Laurent Renia**

Singapore

**Hugh Reyburn**

United Kingdom

**Claude-Agnes Reynaud**

France

**Joanna Reynolds**

United Kingdom

**Paulo Ribolla**

Brazil

**Francesco Ricci**

Italy

**Adam Richards**

United States

**Frank Richards**

United States

**Michael Riscoe**

United States

**Juan Rivero de Aguilar Cachafeiro**

Spain

**Jacob Riveron**

United Kingdom

**Michael Robert**

United States

**Vincent Robert**

France

**Kirk Rockett**

United Kingdom

**Xavier Rodo**

Spain

**Ana Rodriguez**

United States

**Paul Roepe**

United States

**Michel Roger**

Canada

**Stephen Rogerson**

Australia

**Christophe Rogier**

Madagascar

**Sergio Romeo**

Italy

**Philip Rosenthal**

United States

**Amanda Ross**

Switzerland

**Andrea Rother**

South Africa

**Christian Roussilhon**

France

**Mark Rowland**

United Kingdom

**Jose Rubio**

Spain

**Isabel Ruiz**

Colombia

**Esmee Ruizendaal**

Netherlands

**Bruce Russell**

Singapore

**Tanya Russell**

Australia

**Elizeus Rutebemberwa**

Uganda

**Edmund Rutta**

United States

**Anavaj Sakuntabhai**

France

**Joseph Salvino**

United States

**Christina Sammet**

United States

**Rosemary Sang**

Kenya

**Sompob Saralamba**

Thailand

**Demba Sarr**

United States

**Yukita Sato**

Japan

**Huub Savelkoul**

Netherlands

**Henk Schallig**

Netherlands

**David Schellenberg**

United Kingdom

**Patricia Schlagenhauf**

Switzerland

**Yosef Schlein**

Israel

**Kristan Schneider**

Germany

**Alanna Schwartz**

United States

**Jeffrey Scott**

United States

**Thomas Scott**

United States

**Seif Shekalaghe**

Tanzania

**Adelfa E Serrano**

Puerto Rico

**Jeffrey Shaman**

United States

**Sumaira Shams**

Pakistan

**Dennis Shanks**

Australia

**Estifanos Biru Shargie**

Switzerland

**Arun Sharma**

India

**Rajander Sharma**

India

**Shamarina Shohaimi**

Malaysia

**Addmore Shonhai**

South Africa

**Carol Sibley**

United States

**Elisa Sicuri**

Spain

**Inga Siden-Kiamos**

Greece

**Maggy Sikulu**

Australia

**Balbir Singh**

Malaysia

**Neeru Singh**

India

**Photini Sinnis**

United States

**Andre Siqueira**

Brazil

**Jean-Yves Siriez**

France

**Manju Sivasankaran**

India

**Jacek Skarbinski**

United States

**George Skavdis**

Greece

**Ole Skovmand**

France

**Hannah Slater**

United Kingdom

**Brad Sleebs**

Australia

**Larry Slutsker**

United States

**David Smith**

United States

**Thomas Smith**

Switzerland

**Lucy Smith Paintain**

United Kingdom

**Georges Snounou**

France

**Irene Soares**

Brazil

**Claudia Soeiro**

Brazil

**Anne Marit Sponaas**

Norway

**Sanjeeva Srivastava**

India

**Sarah Staedke**

Uganda

**Henry Staines**

United Kingdom

**Peter Starzengruber**

Austria

**Laura Steinhardt**

United States

**Richard Steketee**

France

**Anna-Sofie Stensgaard**

Denmark

**Kasia Stepniewska**

United Kingdom

**Jose Stoute**

United States

**Daniel Strickman**

United States

**Gary Strobel**

United States

**Erin Stuckey**

Switzerland

**Hugh Sturrock**

United States

**Martha Cecilia Suarez-Mutis**

Brazil

**David Sullivan**

United States

**Avadhesha Surolia**

India

**Awalludin Sutamihardja**

Indonesia

**James Sutcliffe**

Canada

**Colin Sutherland**

United Kingdom

**Patrick Sutton**

United States

**Gote Swedberg**

Sweden

**Patricia Tabernero**

United Kingdom

**Carlos Tadokoro**

Brazil

**Harry Tagbor**

Ghana

**Ambrose Talisuna**

Kenya

**Paul Tambyah**

Singapore

**Kathrine Tan**

United States

**Marcel Tanner**

Switzerland

**Geoff Targett**

United Kingdom

**Joel Tarning**

Thailand

**Andrew Tatem**

United Kingdom

**Nicolas Taudon**

France

**WRJ Taylor**

Thailand

**Diane Taylor**

United States

**Steve Taylor**

United States

**Fabrizio Tediosi**

Switzerland

**Kevin Tetteh**

United Kingdom

**Michael Theisen**

Denmark

**Matthew Thomas**

United States

**Kamala Thriemer**

Australia

**Phil Thuma**

Zambia

**Julie Thwing**

United States

**Matt Tinsley**

United Kingdom

**Alberto Tobón-Castaño**

Colombia

**Mark Topazian**

United States

**Alessandra Torina**

Italy

**Harold Townson**

United Kingdom

**Yesim Tozan**

United States

**Richard Tren**

United States

**Katharine Trenholme**

Australia

**Abhai Tripathi**

United States

**Marita Troye-Blomberg**

Sweden

**Takafumi Tsuboi**

Japan

**Elizabeth Turner**

United States

**Lucy Tusting**

United Kingdom

**Anna Tynan**

Australia

**Venkatachalam Udhayakumar**

United States

**Juerg Utzinger**

Switzerland

**Innocent Valea**

Burkina Faso

**Neena Valecha**

India

**Andrew Vallely**

Australia

**Wim Van Bortel**

Sweden

**Jean-Pierre Van geertruyden**

Belgium

**Perry van Genderen**

Netherlands

**Jaap van Hellemond**

Netherlands

**Cornelis Van Noorden**

Netherlands

**Wesley Van Voorhis**

United States

**Jodi Vanden Eng**

United States

**Catherine Vaquero**

France

**Charles Vaughan-Williams**

South Africa

**Vijay Veer**

India

**Thirumalaisamy Velavan**

Germany

**Meera Venkatesan**

United States

**Hans Verhoef**

Netherlands

**Lasse Vestergaard**

Philippines

**Ana Margarida Vigário**

Portugal

**Joseph Vinetz**

United States

**Theodoor Visser**

United States

**Sarah Volkman**

United States

**Lorenz von Seidlein**

Thailand

**John Waitumbi**

Kenya

**Larry Walker**

United States

**Edward Walker**

United States

**Judd Walson**

United States

**Nicola Wardrop**

United Kingdom

**David Warhurst**

United Kingdom

**Samuel Wassmer**

United States

**Hilary Watt**

United Kingdom

**Cameron Webb**

Australia

**Rachel Weber**

Tanzania

**Gareth Weedall**

United Kingdom

**Rita Wegmüller**

Gambia

**Daniel Weiss**

United Kingdom

**Timothy Wells**

Switzerland

**Edward Wenger**

United States

**Albert Wertheimer**

United States

**Bradley White**

United States

**Lisa White**

Thailand

**Michael White**

United Kingdom

**Maxine Whittaker**

Australia

**Christopher Whitty**

United Kingdom

**Rosanne Wieten**

Netherlands

**Timothy William**

Malaysia

**Holly Ann Williams**

United States

**Kim Williamson**

United States

**Anne Wilson**

United Kingdom

**Mark Wilson**

United States

**Muhwezi Wilson Winstons**

Uganda

**Michael Wimberly**

United States

**Anna Winters**

Zambia

**Elizabeth Winzeler**

United States

**Sergio Wittlin**

Switzerland

**Tamirat Gebru Woldearegai**

Ethiopia

**Lisa Wolfe**

United States

**Charles Wondji**

United Kingdom

**Claire Wong**

New Zealand

**Bayden Wood**

Australia

**Charles Woodrow**

Thailand

**Colin Wright**

United Kingdom

**Gerhard Wunderlich**

Brazil

**Prashant Yadav**

Spain

**Stephanie Yanow**

Canada

**Cedric Yansouni**

Canada

**William Yavo**

Cote d'Ivoire

**Kojo Yeboah-Antwi**

United States

**Adoke Yeka**

Uganda

**Shunmay Yeung**

United Kingdom

**Delenasaw Yewhalaw**

Ethiopia

**Shigeto Yoshida**

Japan

**Joshua Yukich**

United States

**Orlando Zacarias**

Sweden

**Sedigheh Zakeri**

Iran

**Fidel Zavala**

United States

**Hongwei Zhang**

China

**Tongyan Zhao**

China

**Xiao-Nong Zhou**

China

**Peter Zimmerman**

United States

**Thomas Zoller**

Germany

**Dejan Zurovac**

Kenya

